# The CENP-O complex requirement varies among different cell types

**DOI:** 10.1007/s10577-014-9404-1

**Published:** 2014-01-31

**Authors:** Naoko Kagawa, Tetsuya Hori, Yuko Hoki, Osamu Hosoya, Kimiko Tsutsui, Yumiko Saga, Takashi Sado, Tatsuo Fukagawa

**Affiliations:** 1 Department of Molecular Genetics, National Institute of Genetics and The Graduate University for Advanced Studies, Mishima, Shizuoka 411-8540 Japan; 2 Division of Epigenomics and Development, Medical Institute of Bioregulation, Kyushu University, 3-1-1 Maidashi, Higashi-ku, Fukuoka, Fukuoka 812-8582 Japan; 3 Department of Neurogenomics, Graduate School of Medicine, Dentistry and Pharmaceutical Sciences, Okayama University, Okayama, 700-8558 Japan; 4 Division of Mammalian Development, National Institute of Genetics and The Graduate University for Advanced Studies, Mishima, Shizuoka 411-8540 Japan; 5 National Institute of Genetics, Mishima, Shizuoka 411-8540 Japan

**Keywords:** Centromere, Kinetochore, CENP-O complex proteins

## Abstract

CENP-U (CENP-50) is a component of the CENP-O complex, which includes CENP-O, CENP-P, CENP-Q, CENP-R, and CENP-U and is constitutively localized at kinetochores throughout the cell cycle in vertebrates. Although CENP-U deficiency results in some mitotic defects in chicken DT40 cells, CENP-U-deficient chicken DT40 cells are viable. To examine the functional roles of CENP-U in an organism-dependent context, we generated CENP-U-deficient mice. The CENP-U-deficient mice died during early embryogenesis (approximately E7.5). Thus, conditional CENP-U-deficient mouse ES cells were generated to analyze CENP-U-deficient phenotypes at the cell level. When CENP-U was disrupted in the mouse ES cells, all CENP-O complex proteins disappeared from kinetochores. In contrast, other kinetochore proteins were recruited in CENP-U-deficient mouse ES cells as CENP-U-deficient DT40 cells. However, the CENP-U-deficient ES cells died after exhibiting abnormal mitotic behavior. Although CENP-U was essential for cell viability during mouse early embryogenesis, CENP-U-deficient mouse embryonic fibroblast cells were viable, similar to the DT40 cells. Thus, although both DT40 and ES cells with CENP-U deficiency have similar mitotic defects, cellular responses to mitotic defects vary among different cell types.

**Electronic supplementary material:**

The online version of this article (doi:10.1007/s10577-014-9404-1) contains supplementary material, which is available to authorized users.

## Introduction

Accurate chromosome segregation during mitosis is essential for the correct transmission of genetic material. To promote faithful chromosome segregation, the formation of a kinetochore on centromeric DNA is essential (Cheeseman and Desai 2008; Perpelescu and Fukagawa 2011; Hori and Fukagawa 2012). Multiple kinetochore proteins have been recently identified in vertebrate cells using combinations of various approaches (Foltz et al. 2006; Okada et al. 2006; Izuta et al. 2006; Hori et al. 2008a; Amano et al. 2009; Meraldi et al. 2006; Cheeseman and Desai 2008). These studies revealed that a constitutive centromere-associated network (CCAN) of proteins was associated with centromeres throughout the cell cycle and provided a platform for the formation of a functional kinetochore during mitosis.

We have shown that CCAN proteins are divided into several subcomplexes (Okada et al. 2006; Hori et al. 2008b; Perpelescu and Fukagawa 2011). In this study, we focused on the CENP-O complex, which includes CENP-O, CENP-P, CENP-Q, CENP-R, and CENP-U (CENP-50). We previously generated chicken DT40 cell lines deficient of each of these proteins using a gene knockout (KO) approach and found that the kinetochore localizations of CENP-O, CENP-P, CENP-Q, and CENP-U were interdependent and that CENP-R localization occurred downstream of these four other proteins, on the basis of analyses of these CENP-O complex-deficient cell lines (Minoshima et al. 2005; Hori et al. 2008b). In addition, we demonstrated that kinetochore localization of CENP-H-associated proteins occurred upstream of the CENP-O complex in chicken DT40 cells (Minoshima et al. 2005; Hori et al. 2008b). Consistent with these observations, coexpression of these proteins in *Escherichia coli* cells showed that CENP-O, CENP-P, CENP-Q, and CENP-U proteins formed a stable complex that could associate with CENP-R (Hori et al. 2008b). Thus, we concluded that CENP-O, CENP-P, CENP-Q, and CENP-U proteins formed a stable complex and that CENP-R functioned downstream of these four proteins. Based on immunofluorescence analyses, the CENP-O complex functioned downstream of CENP-H-associated CCAN proteins in chicken DT40 cells (Minoshima et al. 2005; Hori et al. 2008b).

Chicken DT40 cells with KO of each CENP-O complex protein were viable, although they exhibited subtle mitotic defects (Minoshima et al. 2005; Okada et al. 2006; Hori et al. 2008b). However, the requirement for the CENP-O complex proteins for the viability of other cell types remained uncertain. Thus, it is essential to determine the roles of the CENP-O complex proteins in other cell types. To examine the functional roles of the CENP-O complex in an organism-dependent context, we focused on CENP-U, because CENP-U was firstly identified as a component of the CENP-O complex (Minoshima et al. 2005; Hori et al. 2008b). We generated CENP-U-deficient mice and found that these mice died during early embryogenesis (approximately E7.5). To analyze CENP-U-deficient phenotypes in the mouse ES and mouse embryonic fibroblast (MEF) cells, we also generated CENP-U-deficient ES and MEF cells. Although kinetochore organization in the CENP-U-deficient ES cells was similar to that in CENP-U-deficient DT40 cells, the CENP-U-deficient ES cells died after they exhibited abnormal mitotic behavior. In contrast, CENP-U-deficient MEF cells were viable, similar to the DT40 cells. Thus, we conclude that although both DT40 and ES cells with CENP-U deficiency have similar mitotic defects, the cellular responses to these mitotic defects vary among different cell types.

## Results

### CENP-U is essential for the progression of mouse development

**Fig. 1:**
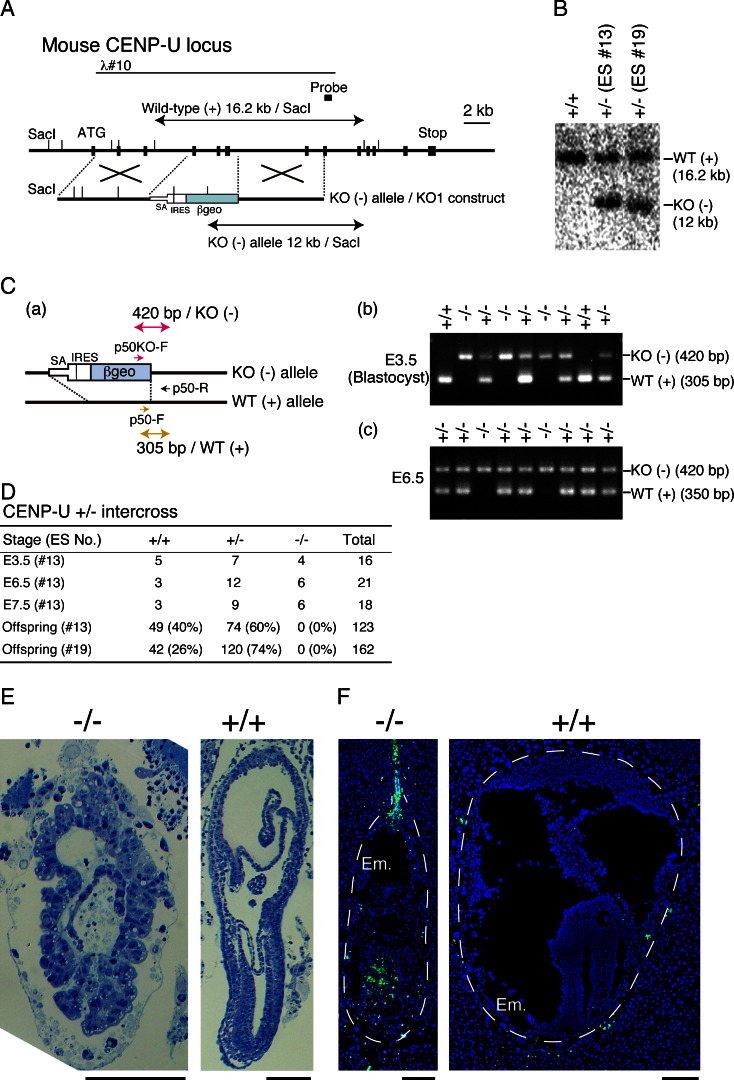
CENP-U is essential for mouse embryogenesis **a** Genomic region of the mouse CENP-U locus and a gene disruption construct. *Black boxes* indicate the positions of exons. Sac I restriction sites are shown. The position of the probe used for Southern hybridization is indicated. A novel 12-kb Sac I fragment hybridized to the probe, if targeted integration of the construct occurred. **b** Restriction analysis of genomic DNAs with targeted integration of the CENP-U disruption construct. Genomic DNAs from wild-type ES cells and two clones (#13 and #19) after targeting (+/−) were analyzed by Southern hybridization using the probe indicated in (A). In #13 and #19, a novel 12-kb Sac I fragment was detected. **c** PCR genotyping of embryonic DNA from mice of CENP-U^+/−^ heterozygous intercrosses. **a** Primer design, **b** PCR results with DNA from E3.5, and **c** PCR results with DNA from E6.5. **d** Genotyping of CENP-U^+/−^ intercross mice at each stage. **e** Serial section analysis of E7.5 embryos from wild-type or CENP-U^−/−^ mice. Sections were stained with toluidine blue. Scale bar, 100 μm. **f** DAPI (*blue*) and TUNEL (*green*) staining of E7.5 embryos from the wild-type or CENP-U^−/−^ mice. Scale bar, 200 μm. An embryo (*Em*.) is outlined by a *dashed line*. TUNEL positive cells are enriched in the ^−/−^ embryo

To disrupt the mouse CENP-U gene in ES cells, we used a promoter-less targeting construct to obtain a high level of homologous recombination. If a targeting reaction occurred, a neomycin resistance gene would be expressed under the control of the CENP-U promoter and exons 4 to 6 of the CENP-U gene would be deleted (Fig. 1a). We isolated several 129/Sv-derived ES cell clones with disrupted a CENP-U allele and confirmed targeted disruption by Southern blot analysis (Fig. 1b). ES cells with the CENP-U-disrupted allele were injected into C57BL/6 blastocysts, and the resulting chimerical mice were backcrossed with the wild-type C57BL/6 mice to generate CENP-U^+/−^ heterozygous mice. We designed a PCR primer set to distinguish between wild-type and disrupted alleles and performed genotyping analysis of embryonic cells from CENP-U^+/−^ heterozygous intercrosses (Fig. 1c).

We could detect cells with the CENP-U^−/−^ genotype among E3.5 and E6.5 embryos. However, no homozygous null mice were obtained from among a total of 285 live births from the CENP-U^+/−^ heterozygous intercrosses (Fig. 1c, d). Wild-type and CENP-U^+/−^ heterozygous mice were born at the expected frequencies, which suggested that the CENP-U^−/−^ mice were embryonic lethal. We also analyzed embryos with CENP-U^−/−^ alleles at E7.5 and found that embryos from the CENP-U^−/−^ mice were smaller than those from the wild-type or heterozygous mice (Fig. 1e, f). In addition, many apoptotic cells were detected among CENP-U^−/−^ cells (Fig. 1e, f), which indicated that CENP-U-deficient embryos stopped proliferating before E7.5 and died.

Although CENP-U and other CENP-U-associated proteins are dispensable in chicken DT40 cells (Minoshima et al. 2005; Okada et al. 2006; Hori et al. 2008b), the results indicated that CENP-U was essential for cell proliferation during mouse development.

### CENP-U is essential for the viability of mouse ES cells

**Fig. 2:**
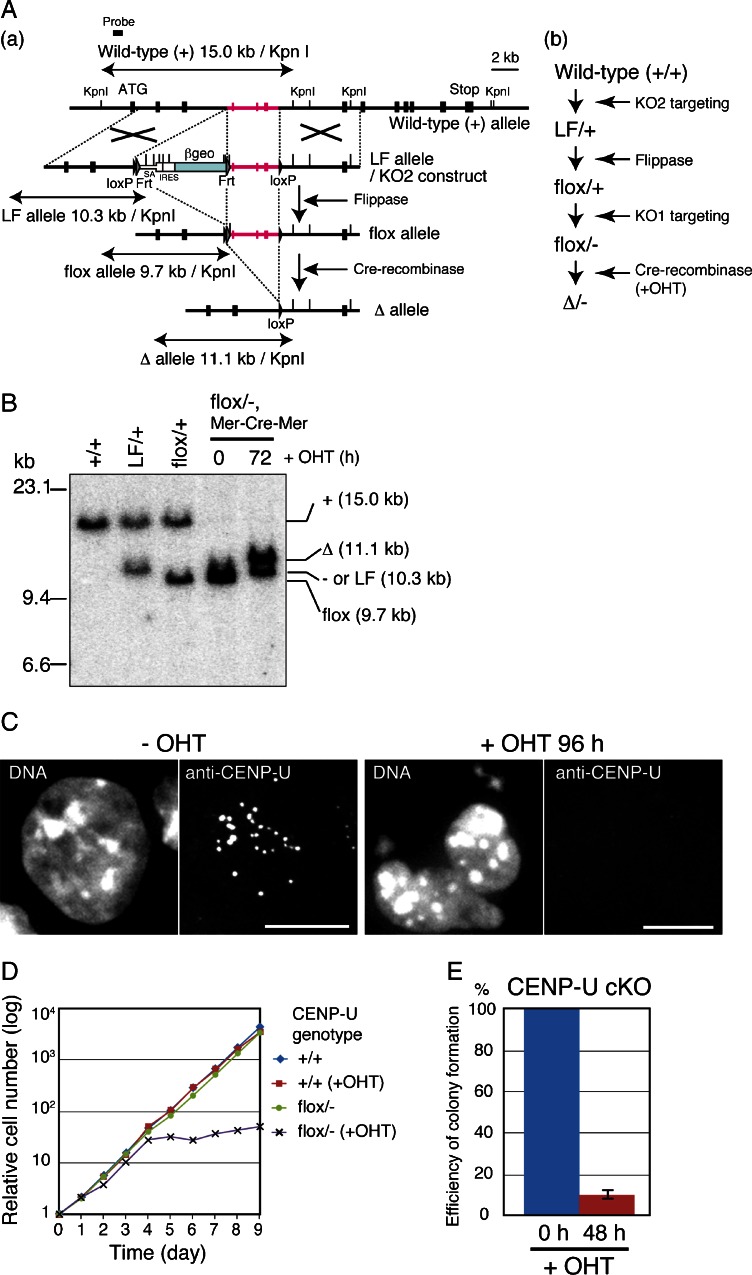
CENP-U is essential for the viability of mouse ES cells. **a** Strategy used for generating conditional CENP-U-deficient ES cells. (*a*) Constructs used for sequential gene targeting. (*b*) Targeting strategy. Mouse ES cells with CENP-U^flox/−^ alleles were obtained by sequential gene targeting. When OHT was added to ES cells with CENP-U^flox/−^ alleles to activate Cre-recombinase (Mer-Cre-Mer), homologous recombination occurred between two loxP sites and ES cells with the CENP-U^Δ/−^ allele were conditionally obtained. **b** Genotype analysis of conditional CENP-U-deficient ES cells. After adding OHT to ES cells with CENP-U^flox/−^ alleles, recombination occurred between two loxP sites. **c** Immunofluorescence analysis of ES cells with CENP-U^flox/−^ alleles before or after adding OHT with anti-CENP-U antibody. Scale bar, 10 μm. **d** Cell growth analysis for ES cells with CENP-U^flox/−^ alleles in the presence or absence of OHT. **e** Colony formation efficiency for ES cells with CENP-U^flox/−^ alleles in the presence or absence of OHT

Because the CENP-U-deficient mice were embryonic lethal, we attempted to determine why these mice had died during early embryogenesis. To examine CENP-U requirements for viability at the ES cell level, we generated conditional CENP-U-deficient mouse ES cells by a gene targeting approach. The strategy used to generate conditional CENP-U-deficient ES cells is shown in Fig. 2a, b. By sequential gene targeting, we finally generated mouse ES cells with CENP-U^flox/−^ alleles (CENP-U^flox/−^ ES cells) (Fig. 2b).

Because we had integrated a Mer-Cre-Mer construct in this cell line, we could conditionally remove the CENP-U gene by adding 4-hydroxytamoxfen (OHT) to activate Cre-recombinase-mediated homologous recombination between two loxP sites (CENP-U^∆/−^ ES cells). We confirmed that recombination had occurred between two loxP sites after adding OHT to these cells (Fig. 2b) and found that the punctate signals for CENP-U had disappeared in the CENP-U^∆/−^ ES cells, on the basis of immunofluorescence analysis using anti-CENP-U antibody (Fig. 2c).

Then, we investigated cell growth and colony formation efficiency of the CENP-U^flox/−^ ES cells after adding OHT. As shown in Fig. 2d, the CENP-U^flox/−^ ES cells stopped growing 4 days after adding OHT and finally died. This growth behavior was in contrast to that of CENP-U-deficient DT40 cells (Minoshima et al. 2005; Hori et al. 2008b). In addition, the colony formation efficiency of the CENP-U^flox/−^ ES cells at 48 h was significantly reduced after adding OHT (Fig. 2e).

### Localizations of CENP-O complex proteins are interdependent in mouse ES cells

**Fig. 3:**
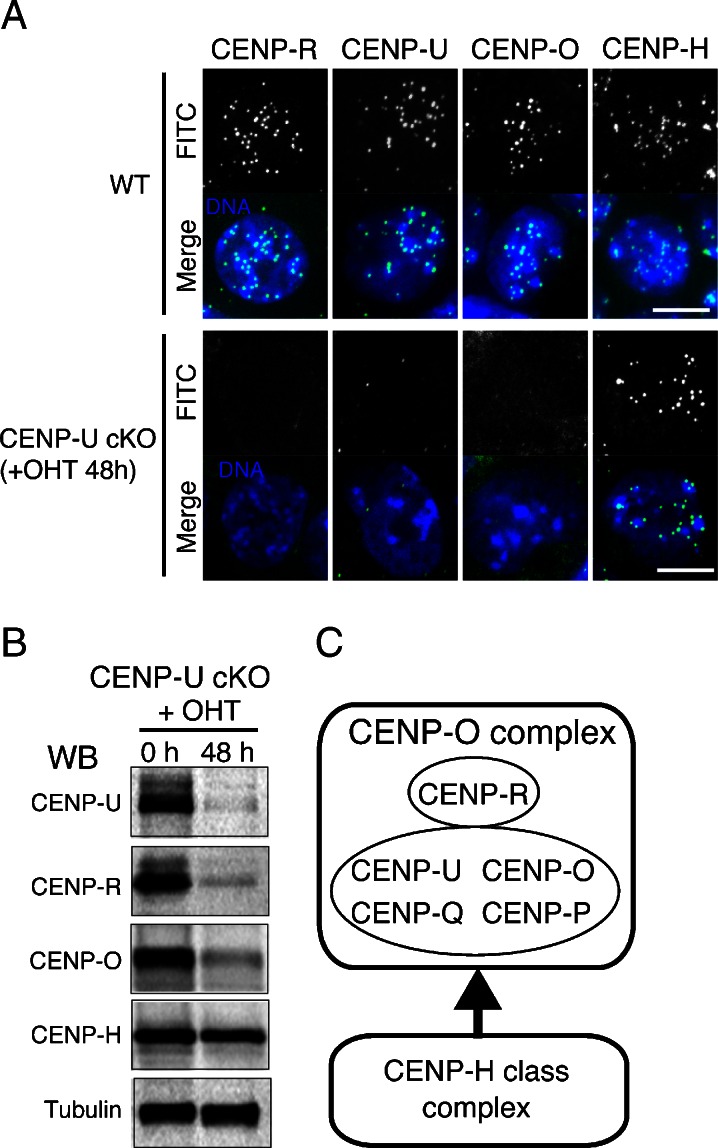
Localizations of centromeric proteins in CENP-U and CENP-R-deficient ES cells. **a** Immunofluorescence analysis of wild-type, CENP-U-deficient ES cells (+OHT 48 h) using antibodies against CENP-R, CENP-U, CENP-O, and CENP-H. Bar, 10 μm. **b** Western blot analysis of CENP-U-deficient cells with antibodies against CENP-U, CENP-R, CENP-O, and CENP-H. Anti-tubulin antibody was used as a loading control. The amounts of CENP-U, CENP-R, and CENP-O were decreased in the CENP-U-deficient cells but not those of CENP-H. **c** A summary of the association of CENP-O-class proteins. CENP-O, CENP-P, CENP-Q, and CENP-U form a stable complex. This stable complex functions downstream of CENP-H-associated proteins and upstream of CENP-R

In CENP-U-deficient DT40 cells, CENP-U kinetochore localization occurs downstream of CENP-H and upstream of CENP-R (Hori et al. 2008b). Because the CENP-U requirement for the viability of mouse ES cells was different from that of DT40 cells, the localization hierarchy of centromeric proteins in mouse ES cells may have differed from that in DT40 cells. Thus, we examined the localizations of CENP-H, CENP-O, and CENP-R in the CENP-U-deficient mouse ES cells. CENP-H was clearly detectable in these cells, whereas CENP-O and CENP-R were undetectable (Fig. 3a).

In addition, we investigated the amounts of the CENP-O complex proteins in CENP-U-deficient ES cells using western blot analysis. The CENP-U, CENP-R, and CENP-O protein levels were decreased in the CENP-U-deficient cells, which suggested that these proteins formed a complex similar to that in DT40 cells (Fig. 3b) and that these complexes were unstable in the CENP-U-deficient ES cells.

These results indicated that the localization hierarchy of CENP-O complex proteins in mouse ES cells was similar to that observed in DT40 cells (Fig. 3c) (Hori et al. 2008b). However, the CENP-O complex requirement for the viability of mouse ES cells was different from that of DT40 cells.

### CENP-U-deficient ES cells exhibit mitotic abnormalities

**Fig. 4:**
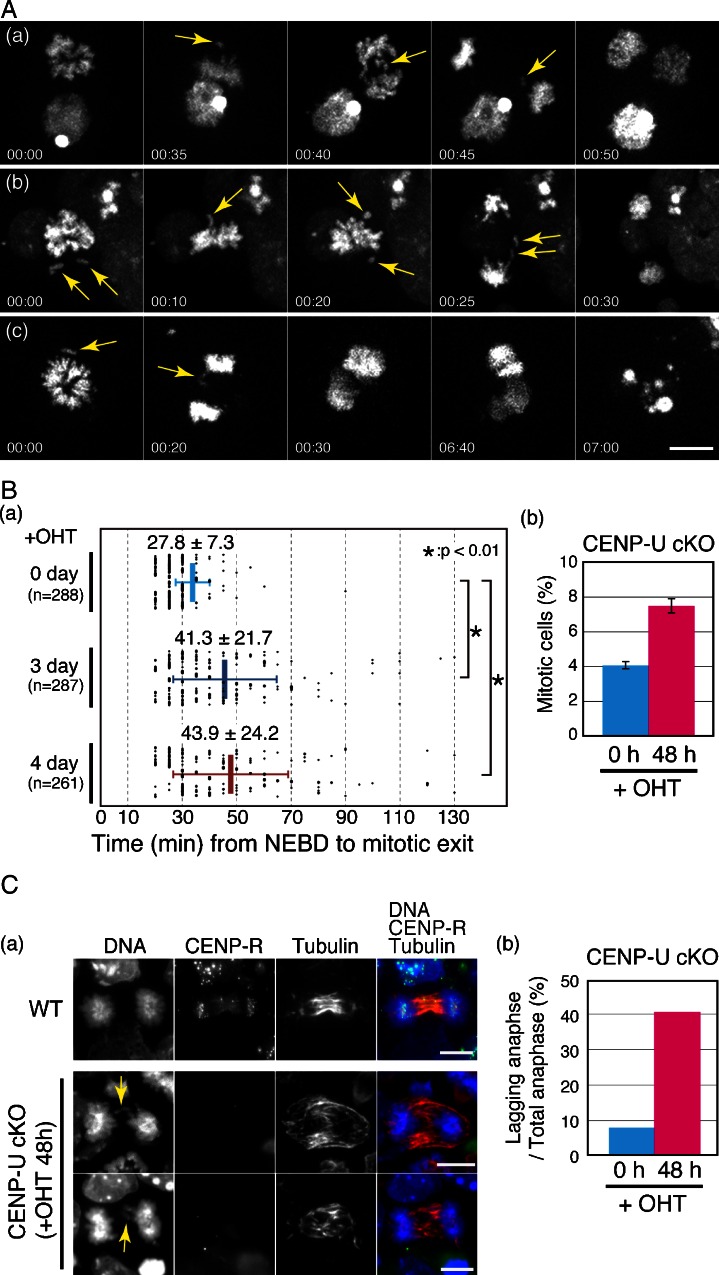
CENP-U-deficient ES cells die during interphase after exhibiting abnormal mitotic behavior. **a** Chromosome dynamics in CENP-U-deficient ES cells as observed by time-lapse microscopy of live cells. Misaligned or lagging chromosomes (*arrows*) were frequently observed. Three individual cells (*a*–*b*) are shown. Scale bar, 10 μm. **b** Quantitation of the time for progression from nuclear envelope breakdown to (NEBD) mitotic exit by ES cells with CENP-U^flox/−^ alleles after adding OHT based on time-lapse microscopy of viable cells. Average times are also shown. *t* test was performed to confirm significance. (*b*) Percentages of mitotic ES cells with CENP-U^flox/−^ alleles were determined in the presence (48 h) or absence of OHT. Fixed cells were used for these determinations. **c** Percentages of cells with lagging chromosomes (*arrows*). ES cells with CENP-U^flox/−^ alleles were fixed and stained with anti-CENP-R and tubulin antibodies in the presence (48 h) or absence of OHT. Scale bar, 10 μm. (*b*) Percentages of cells with lagging chromosomes

To examine the mitotic behavior of the CENP-U-deficient ES cells, we monitored mitotic progression by the CENP-U^flox/−^ ES cells using live cell imaging after adding OHT. For live cell imaging, we prepared CENP-U^flox/−^ ES cells that expressed histone H2B-RFP. Typical images and movies are shown in Fig. 4a and supplemental movies S1–3.

We found that numerous cells exhibited prolonged mitosis (Fig. 4b). Control cells completed mitosis within 28 ± 8 min, whereas the CENP-U-deficient ES cells (4 days after adding OHT to CENP-U^flox/−^ ES cells) completed mitosis within 44 ± 24 min. After this prolonged mitosis, these CENP-U-deficient cells entered anaphase and exited mitosis. During anaphase, we frequently observed lagging chromosomes (Fig. 4c); more than 40 % of the CENP-U-deficient cells contained lagging chromosomes (Fig. 4c).

Although CENP-U-deficient ES cells exhibited abnormal mitotic behavior, these cells finally entered into the next interphase. However, many of these cells suddenly died during interphase after exhibiting abnormal mitotic behavior (Fig. 4a–c and supplemental movie S3).

### CENP-U is dispensable in MEF and chicken DT40 cells

CENP-U was essential for cell growth by early embryonic mouse cells and undifferentiated mouse ES cells. In contrast, CENP-U was dispensable in chicken B cell-derived DT40 cells. Because the localization hierarchy of the CENP-O complex proteins was conserved for both chicken DT40 and mouse ES cells (Fig. 3), the CENP-O complex requirement for cell viability may vary among different cell types or species.

**Fig. 5:**
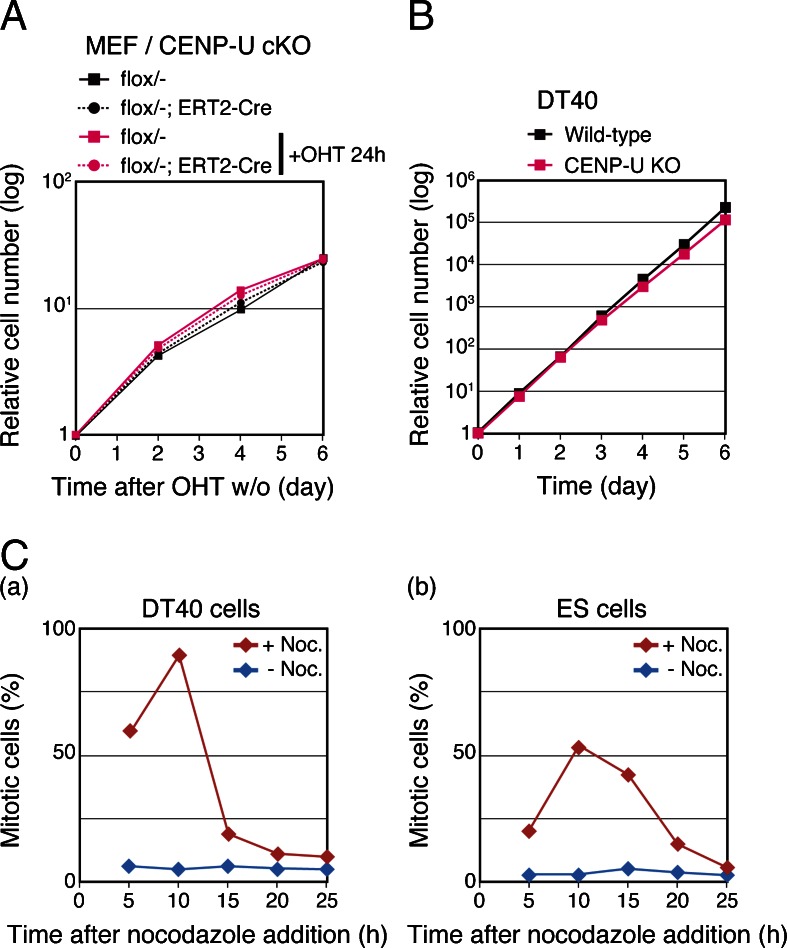
CENP-U is dispensable in MEF cells and chicken DT40 cells. **a** Growth curves for CENP-U-deficient MEF cells. The MEF cells were generated from mice with CENP-U^flox/−^ alleles and ERT2-Cre. The CENP-U gene was removed by adding OHT. **b** Growth curve for CENP-U-deficient DT40 cells. **c** Mitotic checkpoint responses in chicken DT40 (**c**-(*a*)) and mouse ES (**c**-(*b*)) cells. Nocodazole (500 ng/ml) was added to DT40 or mouse ES cell cultures for the indicated times. Mitotic indexes were determined at each time point

To test a CENP-U requirement in other mouse cells, we examined cell growth using CENP-U-deficient MEF cells. To obtain the CENP-U-deficient MEF cells, we generated mice with CENP-U^flox/−^ alleles by crossing CENP-U^+/−^ and CENP-U^flox/+^ mice (Fig. S1). We established MEF cells from the embryos of the CENP-U^flox/−^ mice. Then, we conditionally expressed Cre-recombinase in theses MEF cells to obtain the CENP-U-deficient MEF cells (Fig. 5a).

Unlike the CENP-U-deficient ES cells, the CENP-U-deficient MEF cells grew well, similar to the control MEF cells (Fig. 5a), which indicated that CENP-U was dispensable in MEF cells as in the chicken DT40 cells (Fig. 5b). Thus, our data indicate that the CENP-U requirement for cell viability varied among chicken DT40, mouse ES, and MEF cells.

The kinetochore structure in the CENP-U-deficient mouse ES cells was similar to that in the CENP-U-deficient chicken DT40 cells (Fig. 3). However, we frequently observed lagging chromosomes during anaphase in the CENP-U-deficient mouse ES cells (Fig. 4), compared with that in the chicken DT40 cells. We hypothesized that a mitotic checkpoint response in the mouse ES cells may differ from that in the chicken DT40 cells.

To examine the mitotic checkpoint response in the ES and DT40 cells, we determined the percentages of mitotic cells in the presence of the mitotic spindle poison nocodazole (500 ng/μl). As shown in Fig. 5c, more than 90 % of the DT40 cells were mitotic arrested, but fewer than 60 % of the ES cells were mitotic arrested after 10 h of treatment with nocodazole. Even when we examined various concentrations of nocodazole and treated nocodazole for longer time, we did not find conditions by which more than 60 % of the ES cells were mitotic arrested. This suggested that the ES cells readily progressed to the next stages even in the presence of nocodazole. In consideration of these results, we propose that checkpoint activity in ES cells is weaker than that in DT40 cells and that CENP-U-deficient ES cells readily go through the next interphase after their abnormal mitosis and ultimately die during interphase (Fig. 4a).

## Discussion

CENP-U forms a CENP-O complex with CENP-O, CENP-P, CENP-Q, and CENP-R in chicken DT40 cells (Hori et al. 2008b). The kinetochore localizations of the CENP-O complex proteins are interdependent in vivo, and each chicken recombinant protein forms a stable complex in vitro. Consistent with our analyses, recent microscopic observations suggested that human CENP-O complex proteins assembled at kinetochores to form stable complexes in human cells (Eskat et al. 2012). Polo-like kinase 1 (Plk-1) binds to both chicken and human CENP-U and phosphorylates this protein (Kang et al. 2011; Hori et al. 2008b). Although CENP-U phosphorylation by Plk-1 seems to contribute to CENP-U stability in human cells, DT40 cells, in which wild-type CENP-U is replaced with phospho-dead mutant CENP-U, exhibit some mitotic defects. However, these DT40 cells are still viable (Hori et al. 2008b).

In addition, CENP-U or CENP-Q appears to directly bind to microtubules in human cells (Amaro et al. 2010; Hua et al. 2011), However, the CENP-O complex proteins may not be major components for microtubule binding, at least in the DT40 cells, because chromosome segregation can occur in the CENP-U-deficient DT40 cells. We previously proposed that the CENP-O complex was crucial for recovery from spindle damage but that it was not essential for cell viability under standard culture conditions for the DT40 cells.

In this study, we demonstrated that CENP-U-deficient mice died and their ES cells also died during interphase after exhibiting abnormal mitotic behavior. As CENP-U-deficient mouse ES cells displayed abnormal mitotic behavior, we speculated that CENP-U-deficient ES cells may have acquired abnormal chromosome rearrangements or damages after abnormal mitosis cycles and these abnormalities may have caused cell death during interphase.

Interestingly, CENP-U was dispensable for cell growth by the MEF and DT40 cells. Because the kinetochore structure in the CENP-U-deficient ES cells was similar to that in the CENP-U-deficient DT40 cells (Fig. 3), the functional roles of CENP-U may be conserved. Therefore, we propose that cellular responses to mitotic defects caused by CENP-U deficiency vary among different cell types.

Kinetochores without the CENP-O complex were not fully functional in both mouse ES and chicken DT40 cells as we observed some mitotic defects among numerous cells. However, these defects may be repaired in the DT40 or MEF cells during prolonged mitosis by activating a mitotic checkpoint(s). In contrast, the CENP-U-deficient ES cells may enter the next interphase without repairing their mitotic defects because of the weak checkpoint activity in these cells. Interphase ES cells after abnormal mitosis may have induced chromosome damages, which would cause cell death. Consistent with our observations, mouse and human ES cells frequently re-enter a polyploid cell cycle after escaping mitotic arrest mediated by the spindle checkpoint (Mantel et al. 2007).

In early embryonic cells and before differentiation, cell cycle progression must be strictly regulated for their timely differentiation. Therefore, the checkpoint activity may be weak during early embryogenesis and there may not be sufficient time to repair some mitotic defects. Subsequently, the cells might progress to the next stage of the cell cycle after some mitotic defects arise and ultimately die. In addition, ES cells do not undergo apoptosis after prolonged mitosis and enter the next cell cycle stage (Mantel et al. 2007).

However, if embryonic cells acquire genomic damage and differentiate into specific cell types, this would pose a risk for proper development. Therefore, these abnormal embryonic cells must die. A kinetochore structure that lacks the CENP-O complex is not fully functional although these kinetochores may allow some differentiated cells to survive, such as DT40 or MEF cells, but not undifferentiated embryonic cells.

Although we proposed that mitotic checkpoint responses are various among chicken DT40, mouse ES, and MEF cells, there are alternative explanations why CENP-U deficiency causes different cell viabilities among different cell types. In addition, it is possible to explain that CENP-U-deficient ES cells went to the next interphase not due to a weak checkpoint response. For example, MEF cells may be transformed through multiple passages in cell cultures. In any cases, we would like to emphasize that our various knockout cell lines for CENP-U provide useful resources to understand mechanisms for requirement of CENP-O complex proteins among different cell types.

## Materials and methods

### Cell culture

Mouse ES cells were cultured in Dulbecco’s modified medium supplemented with 15 % fetal calf serum, 0.1 mM non-essential amino acids solution (GIBCO), 500 or 1,000 U/ml LIF (ESGRO) (Millipore), 100 μM beta-mercaptoethanol, and penicillin–streptomycin (GIBCO). ES cells were cultured on mitotically inactivated embryonic feeder cells in gelatin-coated dishes. The ES cells (129/terSV/J1ES) and G418-resistant feeder cells were used to generate CENP-U-deficient mice.

Mouse embryonic fibroblast (MEF) cells were prepared from day E14.5 embryos from wild-type or CENP-U^flox/−^ mice. The MEF cells were cultured in MEF medium and were used for growth rate analysis or immunocytochemistry after the third passage.

### Mouse strains

All mice were maintained under the guidelines for animal experiments at the National Institute of Genetics. The C57BL/6 strain was used as recipients for targeted ES cells and as the background strain for all mutants used in this study. After establishing CENP-U^+/−^ heterozygous mice, CENP-U^−/−^ mice were generated by CENP-U^+/−^ heterozygous intercrosses. CENP-U^flox/−^ mice were also generated to analyze CENP-U deficiency in the MEF cells. For PCR genotyping, mouse tail DNA was extracted and 1 μl of the crude extract was used as a PCR template.

### Immunofluorescence analysis

Mouse ES and MEF cells were placed on slides using a cytocentrifuge and fixed in 3 % paraformaldehyde in 250 mM HEPES at room temperature for 15 min or with chilled methanol at −20 °C for 20 min. Then, samples were permeabilized with 0.5 % NP-40 in PBS at room temperature for 15 min and incubated with an appropriate primary antibody diluted with 0.5 % BSA at 37 °C for 1 h or at 4 °C overnight. After washing, FITC- or Cy3-conjugated secondary antibodies diluted with 0.5 % BSA/PBS were used. Nuclei or chromosomes were counterstained with 4′,6-diamidino-2-phenylindole (DAPI; 0.2 μg/ml) in Vectashield antifade (Vector Laboratories). Immunofluorescence images were acquired using a cooled EM CCD camera (QuantEM, Roper Scientific) mounted on an Olympus IX71 inverted microscope with a 100× objective lens together with a filter wheel and a DSU confocal system. Z-section images were acquired at 0.2-μm intervals and analyzed using Metamorph software (Molecular Device).

### Live cell imaging

For live cell imaging, a histone H2B-RFP plasmid was transfected into CENP-U-deficient ES cells to visualize nuclei and chromosomes. Viable cells were observed using a Confocal Scanner Box, Cell Voyager CV1000 (Yokogawa) with an oil immersion objective lens (PlanApo 60×, NA = 1.40), and the temperature was maintained at 38 °C. Time-lapse images were recorded at 5-min intervals with an exposure time of 0.2–0.3 s. Z-sections (*n* = 15–25) for GFP signals were acquired at 0.3-μm steps for each time point.

### Cell proliferation and mitotic index assay

ES cells were plated in feeder cell-free six-well plates and cultured. The cells were harvested and counted each day. To assess MEF cell growth curves, MEF cells after the third passage were incubated with 100 μM of OHT for 24 h and plated in 24-well plates. Every 2 days, the cells were harvested by trypsinization with 0.25 % trypsin/EDTA and cell numbers were counted.

The cells that had attached to the dish surface and cells floating in the culture medium were collected on slides using a cytocentrifuge. After fixation, the cells were costained with anti-H3P10 and anti-CENP-U antibodies. H3P10-positive cells were counted as mitotic cells.

## Electronic supplementary material

Below is the link to the electronic supplementary material.

ESM 1 (MOV 139 kb)

ESM 2 (MOV 161 kb)

ESM 3 (MOV 1712 kb)

ESM 4 (PDF 461 kb)

## Abbreviations

CENP: Centromere protein
KO: Knockout
CCAN: Constitutive centromere-associated network
ES cells: Embryonic stem cells
MEF: Mouse embryonic fibroblast
Flox: Flanked by loxP sequence
Mer-Cre-Mer: Murine estrogen receptor-fused Cre recombinase
OHT: 4-hydroxytamoxfen
Plk-1: Polo-like kinase 1
NEBD: Nuclear envelope breakdown
