# Supplementary material for: The CENP-O complex requirement varies among different cell types
Source: Chromosome Res. 2014 Jan 31;22(3):293–303. doi: 10.1007/s10577-014-9404-1 (PMC4129241; doi:10.1007/s10577-014-9404-1)
Supplement: Supplementary file 4 — (PDF 461 kb) [file 10577_2014_9404_MOESM4_ESM.pdf]

Supplementary Figure legends

Supplementary Figure S1. Generating mice with CENP-U conditional alleles

(A) Targeting construct for creating CENP-U^{LF} alleles in mouse ES cells.

(B) Restriction analysis of genomic DNA from ES cells with CENP-U^{LF} alleles. DNA was digested with Hind III, and a novel 10-kb fragment was detected with a probe indicated in (A) if a CENP-U^{LF} allele was created.

(C) Strategy for generating conditional CENP-U-deficient mice. Mice with CENP-U$^{flox/+}$ alleles were generated from ES cells with these alleles. Then, the CENP-U$^{flox/flox}$ mice were obtained by intercrossing with the CENP-U$^{flox/+}$ mice. The ERT2-Cre gene was integrated into the CENP-U$^{+/-}$ mice (Figure 1). Finally, the CENP-U$^{flox/-}$ mice with ERT2-Cre genes were obtained by crosses with the CENP-U$^{flox/flox}$ mice and CENP-U$^{+/-}$ mice with ERT2-Cre genes.

(D) PCR genotyping for distinguishing between wild-type CENP-U (WT), CENP-U-disrupted (KO), and CENP-U flox alleles. (a) Primer design. (b) PCR results with DNA from mice with the indicated alleles.

(E) Immunofluorescence analysis using anti-CENP-U antibody for MEF cells from CENP-U$^{flox/-}$ mice. CENP-U signals were not detected in the presence of OHT. Bar, 10 μm.

Supplementary Movie legends

Supplementary Movie1

Chromosome dynamics in CENP-U-deficient ES cells observed by time-lapse microscopy. Selected images were used in Figure 4A-(a).

Supplementary Movie2

Chromosome dynamics in CENP-U-deficient ES cells observed by time-lapse microscopy. Selected images were used in Figure 4A-(b).

Supplementary Movie3

Chromosome dynamics in CENP-U-deficient ES cells observed by time-lapse microscopy. Selected images were used in Figure 4A-(c).

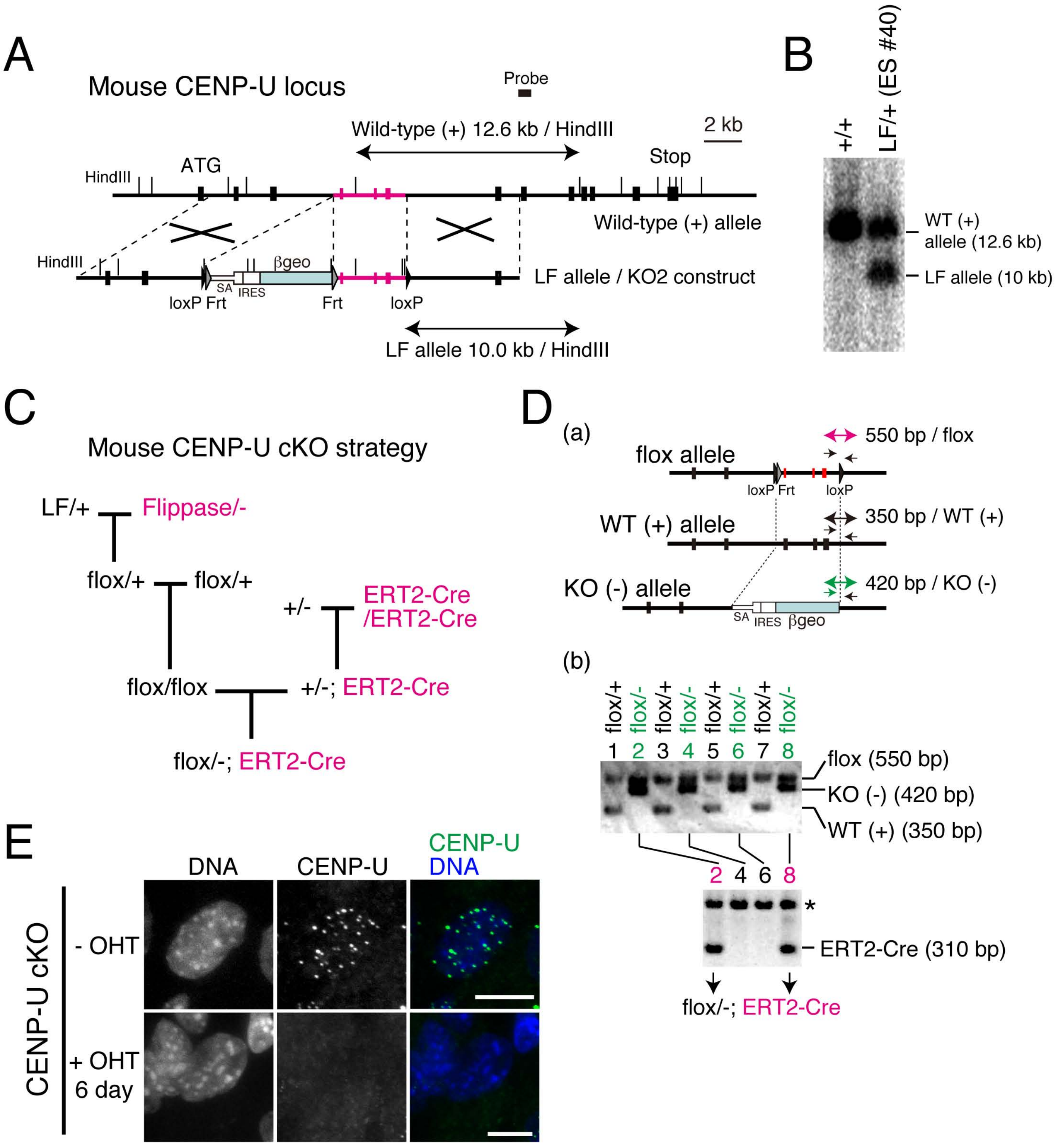
A
Mouse CENP-U locus
Probe
Wild-type (+) 12.6 kb / HindIII
2 kb
HindIII
ATG
Stop
Wild-type (+) allele
HindIII
βgeo
SA
IRES
loxP Frt
Frt
loxP
LF allele / KO2 construct
LF allele 10.0 kb / HindIII
B
+/+
LF/+ (ES #40)
WT (+) allele (12.6 kb)
LF allele (10 kb)
C
Mouse CENP-U cKO strategy
LF/+
Flippase/-
flox/+
flox/+
+/-
ERT2-Cre /ERT2-Cre
flox/flox
+/-; ERT2-Cre
flox/-; ERT2-Cre
D
(a)
flox allele
550 bp / flox
loxP Frt
loxP
WT (+) allele
350 bp / WT (+)
KO (-) allele
420 bp / KO (-)
SA
IRES
βgeo
(b)
flox/+
flox/-
flox/+
flox/-
flox/+
flox/-
flox/+
flox/-
1 2 3 4 5 6 7 8
flox (550 bp)
KO (-) (420 bp)
WT (+) (350 bp)
2 4 6 8
*
ERT2-Cre (310 bp)
flox/-; ERT2-Cre
E
DNA
CENP-U
CENP-U
DNA
CENP-U cKO
- OHT
+ OHT
6 day

Sup. Figure 1
